# Genomic dissection of the most prevalent *Listeria monocytogenes* clone, sequence type ST87, in China

**DOI:** 10.1186/s12864-019-6399-1

**Published:** 2019-12-23

**Authors:** Yan Wang, Lijuan Luo, Qun Li, Hong Wang, Yiqian Wang, Hui Sun, Jianguo Xu, Ruiting Lan, Changyun Ye

**Affiliations:** 1 State Key Laboratory of Infectious Disease Prevention and Control, National Institute for Communicable Disease Control and Prevention, Collaborative Innovation Center for Diagnosis and Treatment of Infectious Diseases, Chinese Center for Disease Control and Prevention, Beijing, 102206 China; 2 School of Biotechnology and Biomolecular Sciences, University of New South Wales, Sydney, NSW 2052 Australia; 3 Zigong Center for Disease Control and Prevention, Zigong, 643000 Sichuan Province China

**Keywords:** *Listeria monocytogenes*, Whole genome sequencing, Genomic comparative analysis, ST87

## Abstract

**Background:**

*Listeria monocytogenes* consists of four lineages that occupy a wide variety of ecological niches. Sequence type (ST) 87 (serotype 1/2b), belonging to lineage I, is one of the most common STs isolated from food products, food associated environments and sporadic listeriosis in China. Here, we performed a comparative genomic analysis of the *L. monocytogenes* ST87 clone by sequencing 71 strains representing a diverse range of sources, different geographical locations and isolation years.

**Results:**

The core genome and pan genome of ST87 contained 2667 genes and 3687 genes respectively. Phylogenetic analysis based on core genome SNPs divided the 71 strains into 10 clades. The clinical strains were distributed among multiple clades. Four clades contained strains from multiple geographic regions and showed high genetic diversity. The major gene content variation of ST87 genomes was due to putative prophages, with eleven hotspots of the genome that harbor prophages. All strains carry an intact CRISRP/Cas system. Two major CRISPR spacer profiles were found which were not clustered phylogenetically. A large plasmid of about 90 Kb, which carried heavy metal resistance genes, was found in 32.4% (23/71) of the strains. All ST87 strains harbored the *Listeria* pathogenicity island (LIPI)-4 and a unique 10-open read frame (ORF) genomic island containing a novel restriction-modification system.

**Conclusion:**

Whole genome sequence analysis of *L. monocytogenes* ST87 enabled a clearer understanding of the population structure and the evolutionary history of ST87 *L. monocytogenes* in China. The novel genetic elements identified may contribute to its virulence and adaptation to different environmental niches. Our findings will be useful for the development of effective strategies for the prevention and treatment of listeriosis caused by this prevalent clone.

## Background

*Listeria monocytogenes* is an important foodborne bacterial pathogen, which is able to switch from a saprotrophic lifestyle to an intracellular pathogenic lifestyle [1]. The bacterium has been isolated from many different natural environments (such as soil, water and plant materials), various food products and food processing environments [2]. *L. monocytogenes* causes listeriosis, which is a severe invasive infection manifesting with sepsis, meningoencephalitis or pregnancy loss, and can be fatal in immunocompromised individuals. Sporadic listeriosis and outbreaks have been reported that were linked to various food products, such as ready-to-eat (RTE) food, raw meat, pasteurized milk, ice cream, fruit, and vegetable [3–5].

*L. monocytogenes* consists of at least four lineages, of which lineages I and II encompass the majority of isolates [6]. Within these two common lineages, the populations have been further divided into sequence types (STs) or clonal complexes (CCs) by multi-locus sequence typing (MLST). Previous studies have found that some clones (STs or CCs) such as CC1, CC2, CC4 and CC6 were strongly associated with sporadic or outbreak listeriosis, whereas some other clones CC9 and CC121 were strongly associated with food product contamination but less with human infections [7]. The difference in prevalence of causing human infections may be associated with difference in virulence. Some clones are known to carry genes conferring higher virulence such as *Listeria* pathogenicity island (LIPI)-3 and LIPI-4 [7, 8], whereas other clones (e.g. ST121) may carry genes better for environmental survival, for example, the stress survival islet (SSI)-1 and SSI-2 [9, 10].

In China, *L. monocytogenes* contamination of food products has been found to be quite extensive [11–13]. However, the prevalence of this pathogen in clinical cases has been seldom reported due to the lack of a good listeriosis surveillance system in China [14]. Our previous studies found that *L. monocytogenes* ST87 was the most common ST in human infections in China and was also commonly found in food products and food associated environments [13–16]. Other studies confirmed our findings [17, 18]. However, ST87 has been rarely reported in other countries with the exception of Spain, where two epidemiological unrelated outbreaks caused by ST87 occurred in 2013 and 2014 [19].

Whole-genome sequencing (WGS) can provide comprehensive knowledge on genetic determinants of the reponse to stress conditions and virulence factors [7, 20]. WGS can elucidate the population structure within a single sequence type or clonal complex more clearly [21, 22]. In this study, we performed WGS analysis on 71 ST87 *L. monocytogenes* strains, which spanned a period of 15 years (from 2001 to 2015), and represented 10 isolation regions (Beijing and Shanghai city, Anhui, Fujian, Guangdong, Henan, Hubei, Jiangsu, Sichuan and Zhejiang province), and seven types of sources (aquatic food, cooked food, poultry, raw meat, vegetables, environment and patients) (Additional file 5: Table S1). We aimed to obtain a clearer understanding of the microevolution of the ST87 clone and putative genetic elements responsible for the adaptation and persistence in enrivonmental niches, and factors facilitating human infections to enable more effective strategies of prevention and control of this foodborne disease in China.

## Results

### The general genomic features of *L. monocytogenes* ST87

**Fig. 1:**
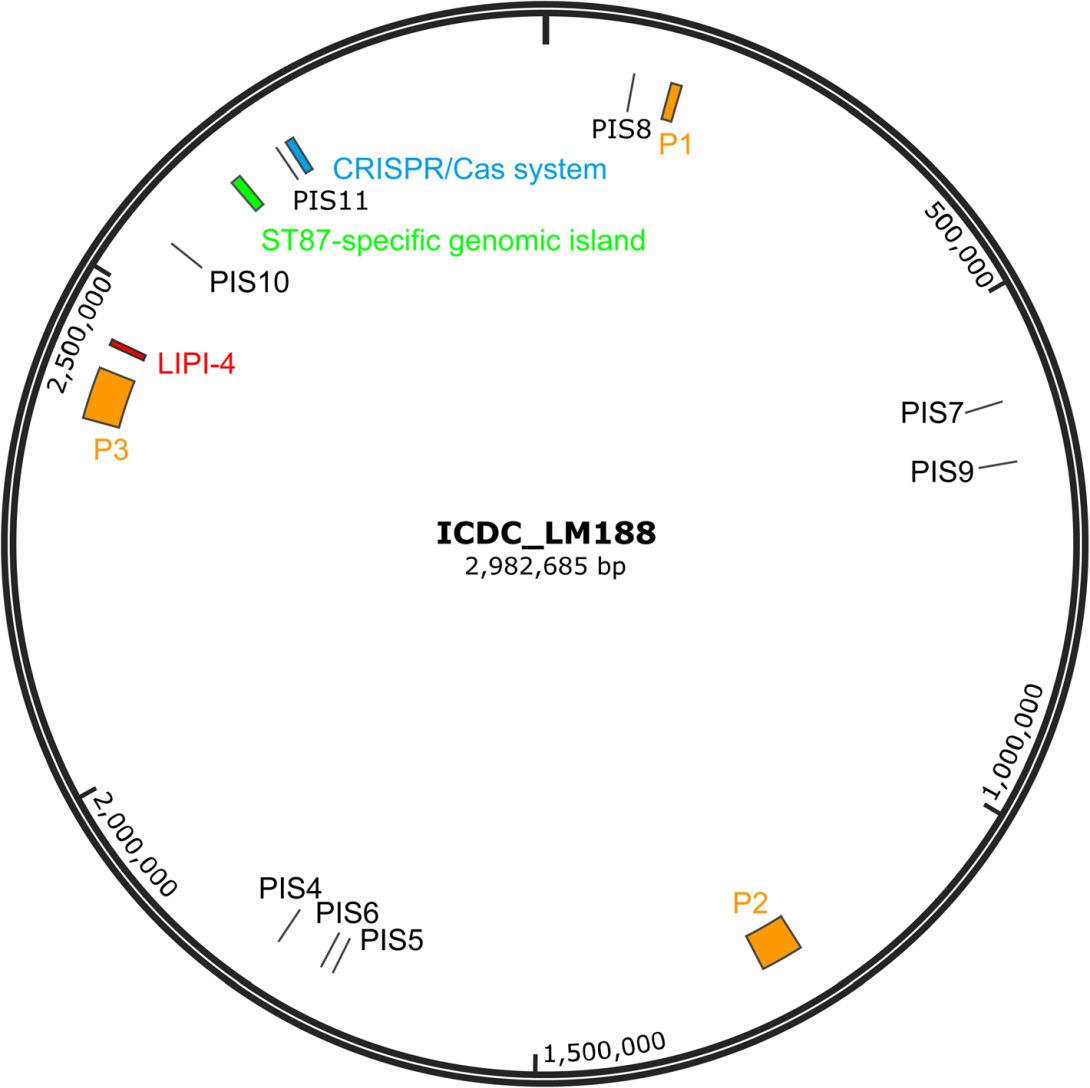
A circular representation of the strain ICDC-LM188 chromosome and the key genetic features of the genome. Putative prophage regions (P1-P3) were marked as orange blocks; LIPI-4, the ST87-specific genomic island and the CRISPR/Cas system were marked as red, green and blue blocks, respectively. The integrated sites of putative prophages (P4 to P11) in other ST87 isolates but not in the strain ICDC-LM188 are labeled as PIS4 to PIS11

To investigate the genomic diversity of *L. monocytogenes* ST87 in China, we sequenced one ST87 strain fully and obtained draft genomes for 70 strains. ST87 strain ICDC-LM188 obtained from a patient was sequenced using Illumina Hiseq 2000 and Applied Biosystems 3730 DNA Analyzer to obtain its complete genome and was used as reference genome in this study. ICDC-LM188 has a 2,982,685 bp single chromosome with G + C content of 37.97% (Fig. 1). The genome contained 2919 genes including 2882 coding sequences (CDS), 67 tRNA genes, six 16S–5S-23S operons and 11 pseudogenes. For the 70 draft genomes, they were sequenced to the depth of 100X coverage on average. The scaffolds of draft genome assemblies varied from 2.8 Mb to 3.1 Mb. The number of predicted genes ranged from 2854 to 3187 (Additional file 6: Table S2). The ST87 pan and core genome contained 3687 genes and 2677 genes respectively. The ST87 core genome is 13.7% larger than the *L. monocytogenes* core genome (2354 genes), while the ST87 pan genome is 15.9% smaller than the species pan genome (4383 genes) [23]. The three most abundant functional categories of the core genes were carbohydrate transport and metabolism (G), transcription (K) and amino acid transport and metabolism (E). While the top three functional categories of the accessory genes were replication, recombination and repair (L), mobilome (X) and transcription (K) (Additional file 1: Figure S1). A large fraction of the gene content (84.0–93.7%) in each strain were core genes.

### Single nucleotide polymorphism (SNP) analysis and phylogenetic analysis

A comparison across all ST87 strains sequenced in this study, using strain ICDC-LM188 as the reference, revealed a total of 5415 SNP positions, of which 1410 (26.0%) were nonsynonymous, 3775 (69.7%) synonymous and 230 (4.3%) in intergenic regions. Of all the SNPs, 644 were located in the core genes (*n* = 478, 17.9%). Interestingly, almost two thirds of the SNPs (*n* = 437) located in the core genes were nonsynonymous, which was very different from the SNPs located on the whole genome. The core genes that harbored SNPs were classified according to the COG database. Based on the predictive function, the largest ratio of nonsynonymous to synonymous mutation occurred in the genes involved in general function prediction only (R), followed by lipid transport and metabolism (I) and inorganic ion transport and metabolism (P) (Table 1).

**Table 1:** Nonsynonymous- and synonymous-SNPs identified within core genes by COG category

COG category	Nonsynonymous	Synonymous	Ratio
Energy production and conversion (C)	14	9	1.56
Cell cycle control, cell division, chromosome partitioning (D)	5	3	1.67
Amino acid transport and metabolism (E)	35	14	2.50
Nucleotide transport and metabolism (F)	10	3	3.33
Carbohydrate transport and metabolism (G)	52	24	2.17
Coenzyme transport and metabolism (H)	14	10	1.40
Lipid transport and metabolism (I)	7	1	7.00
Translation, ribosomal structure and biogenesis (J)	29	9	3.22
Transcription (K)	37	15	2.47
Replication, recombination and repair (L)	46	61	0.75
Cell wall/membrane/envelope biogenesis (M)	20	5	4.00
Cell motility (N)	6	6	1.00
Posttranslational modification, protein turnover, chaperones (O)	16	3	5.33
Inorganic ion transport and metabolism (P)	19	3	6.33
Secondary metabolites biosynthesis, transport and catabolism (Q)	4	2	2.00
General function prediction only (R)	29	3	9.67
Function unknown (S)	30	15	2.00
Signal transduction mechanisms (T)	10	3	3.33
Intracellular trafficking, secretion, and vesicular transport (U)	1	0	
Defense mechanisms (V)	16	3	5.33
Extracellular structures (W)	0	0	
Mobilome: prophages, transposons (X)	1	1	1.00

The top three ratios of nonsynonymous- to synonymous- SNPs harbored core genes were marked as red

**Fig. 2:**
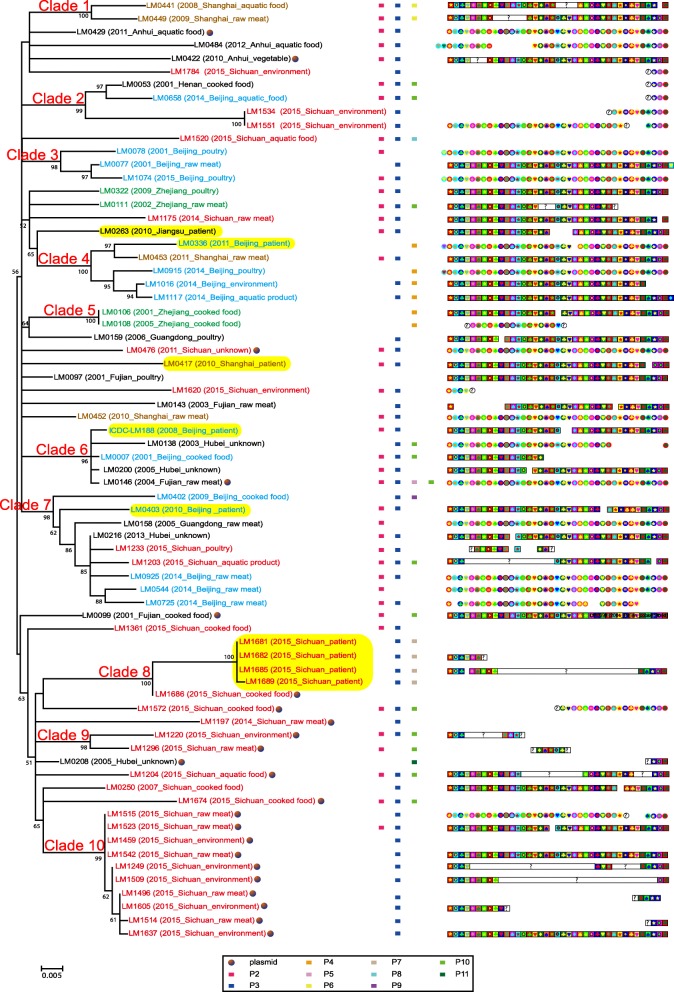
Phylogenetic tree of the 71 ST87 strains. The tree was constructed using the maximum likelihood method based on core genome SNPs. The strains isolated from different regions were marked with different colors (red for Sichuan, blue for Beijing, green for Zhejiang, brown for Shanghai and blank for Fujian, Henan, Hubei and Jiangsu). The strains isolated from clinical cases were highlighted. The strains harbored the plasmid were labeled with a red solid dot. The corresponding putative prophage and the profile of spacers were shown alongside the dendrogram on the right

SNPs present in the core genome were used to infer the phylogenetic relationship of the 71 sequenced ST87 strains using the maximum likelihood method (Fig. 2). Ten clades can be discerned from the SNP-based genome tree with strong bootstrap support. Six of the 10 clades contained strains from the same geographic source. Clade 1, 5 and 9 consisted of only two strains with identical geographic source, but from different years or sources. Clade 8 consisted of five clinical strains in one maternal-fetal case and its closely related strain from a food source. Clade 10 contained 10 strains isolated from a single market in Sichuan in 2015. Four clades contained strains from more than one geographic source and showed higher genetic diversity. However, many branches showed low bootstrap support with a high degree of uncertainty. BEAST analysis indicated that ST87 arose approximately 200 years ago and expanded quickly with a star phylogeny at the base of the tree, which was also reflected on the low bootstrap values of many nodes of the genome tree. To investigate the relationships between the Chinese and non-Chinese ST87 strains, seven ST87 *L. monocytogene* genomes*,* of which six were from the USA and one from Austria*,* were also added to the phylogenetic analysis. The non-Chinese strains were distributed among the Chinese strains with three strains from the USA grouped together as a separate clade (Additional file 2: Figure S2).

### Prophages in ST87 *L. monocytogenes* strains

Using PHAge Search Tool Enhanced Release (PHASTER) online software, three putative prophages have been identified from the complete genome of ICDC-LM188 (Fig. 1). Putative prophage 1 (P1) was 10.7 Kb in size inserted upstream of the 5′-nucleotidase gene (ICDC-LM188 locus tag: A6K41_00700 [*lmo0130* in EGD-e]). P1 has been found as an incomplete prophage previously and was named monocin, which harbored a *lma* operon (*lma*DCBA) and conserved across Lineage I and II strains [21, 22]. Putative prophage 2 (P2) was 43.1 Kb in size with 58 predicted genes, and was inserted downstream of tRNA-Arg-TCT. P2 shared 29 genes with *Listeria* phage LP-101 [24]. Putative prophage 3 (P3) was 52.5 Kb in size with 57 genes, and was inserted into *comK*. P3 shared 34 genes with *Listeria* phage A006. P1 was shared by all sequenced ST87 strains, while P2 and P3 were present in 44 and 62 of the 71 ST87 strains respectively (Fig. 2). It is noteworthy that the genetic contents of P2 and P3 exhibited high diversity among the ST87 *L. monocytogenes* strains.

In addition, another eight putative prophage regions (P4 - P11) were identified in a small proportion of ST87 strains but absent in strain ICDC-LM188. The prophage integration sites of P4 - P11 in the ICDC-LM188 genome were shown in Fig. 1. Prophage P4 was located between the gene encoding FosX/FosE/FosI family fosfomycin resistance thiol transferase (ICDC-LM188 locus tag: A6K41_08870 [*lmo1702* in EGD-e]) and the gene encoding 23S rRNA (uracil-5-)-methyltransferase (RumA, ICDC-LM188 locus tag: A6K41_08875 [*lmo1703* in EGD-e]). Six ST87 strains contained this prophage which was represented by two variants, φLMST87_001 (37.7 Kb) and φLMST87_002 (40.6 Kb). Sequence coverage was 92% with 96% nucleotide identity when the two variants were aligned to each other. φLMST87_001 and φLMST87_002 showed high similarity to the prophage present in *Erysipelothrix* sp. strain LV19, with 95% and 96% nucleotide identity, and 99% and 96% coverage respectively. Both prophages encode an HNH family endonuclease gene and two adjoining DNA methyltransferases genes (adenine- and cytosine-specific, respectively). The R-M system might play a role of excluding other phages when the host encountered exogenous phage infections [25]. They also contained two virulence related proteins. This phage integration site has been reported in a subset of ST204 *L. monocytogenes* strains, which harbored a 56.9 Kb φRNA-MT [21]. However, the gene contents of prophages in ST87 (φLMST87_001 and φLMST87_002) and in ST204 (φRNA-MT) were totally different as the alignment between them showed only 34% and 36% query coverage, respectively. P5, only found in strain LM0146, was integrated into the 3′ end of *tsf* which encodes the elongation factor EF-Ts (ICDC-LM188 locus tag: A6K41_08510 [*lmo1657* in EGD-e]). It showed 96% nucleotide identity with 85% coverage towards the *Listeria* phage B054, and was inserted at the same phage integration site as phage B054. P6, also named as φLMST87_003, is located downstream of the gene encoding methionine adenosyltransferase (MAT, ICDC-LM188 locus tag: A6K41_08565 [*lmo1664* in EGD-e]) and is 43.1 Kb in size. It contained 74 genes. Only two strains, LM0441 and LM0449, harbored φLMST87_003 which showed no similarity with any reported *Listeria* phage. P7 is a putative prophage remnant and was inserted into the upstream of 50S rRNA methyltransferase (ICDC-LM188 locus tag: A6K41_02920 [*lmo0581* in EGD-e]). It was found in four closely related isolates (LM1681, LM1682, LM1685 and LM1689).

We also found four putative prophage regions (P8 - P11) which were typically inserted into chromosomal loci adjacent to tRNA genes, including tRNA-CTT-Lys (ICDC-LM188 locus tag: A6K41_00440, the tRNA-Lys after *lmo0078*), tRNA-CGA-Ser (ICDC-LM188 locus tag: A6K41_03215, the tRNA-Ser after *lmo0670*), tRNA-CCG-Arg (ICDC-LM188 locus tag: A6K41_12845, the tRNA-Arg after *lmo2466*) and tRNA-GGT-Thr (ICDC-LM188 locus tag: A6K41_13515, the tRNA-Thr after *lmo2589*). P10, named φLMST87_004, was assembled into a complete prophage sequence in strain LM1296. It was 43.6 kb in size and has 74 predicted genes. φLMST87_004 showed 90% nucleotide identity with *Listeria* phage A118 with 65% coverage.

### Plasmids in ST87 *L. monocytogenes* strains

**Fig. 3:**
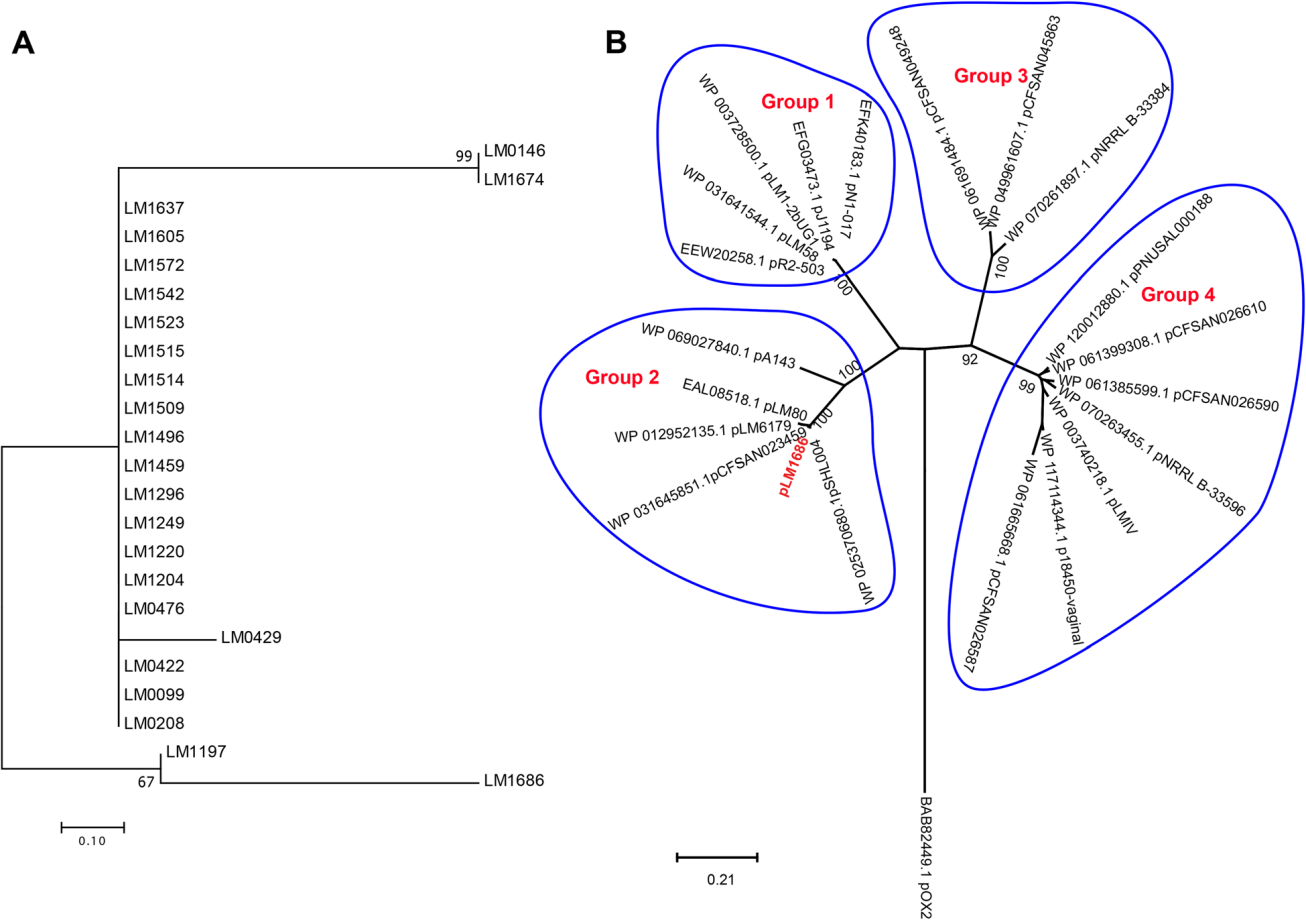
Phylogenetic trees of the plasmids. **a**. Phylogenetic tree of the plasmids of the ST87 strains in this study based on the SNPs in common regions**.** Numbers in % on the nodes are bootstrap values from 1000 replicates. **b**. Phylogenetic tree of pLM1686 and 22 other plasmids in *L. monocytogenes* based on the replication initiation protein, RepA. Numbers in % on the nodes are bootstrap values from 1000 replicates. The RepA of pOX2 from *Bacillus anthracis* was used as an outgroup. pLM1686 was marked in red

We previously identified a plasmid (pLM1686) in strain LM1686 which is homologous to the plasmid from *L. monocytogenes* strain 08–5578 (serotype 1/2a, ST8), with 99% identity in alignments covering 73% of pLM5578. Detailed analysis of pLM1686 has been presented elsewhere [16]. We further determined to which the phylogenetic group pLM1686 from ST87 belonged to among the plasmids in *L. monocytogenes*. We used RepA amino acid sequences from all 22 known plasmids to construct the phylogenetic tree, using the RepA of pOX2 from *Bacillus anthracis* as an outgroup (Fig. 3b). The plasmids clustered into four groups, and pLM1686 belonged to group 2. The plasmid genome contained cadmium resistance *cadAC* genes and also a putative multicopper oxidase gene and copper transporter gene *copB*, but no genes associated with virulence or antimicrobial resistance [16].

pLM1686 is absent in ICDC-LM188. However, plasmids very similar to pLM1686 were found in 22 other ST87 strains sequenced in this study. All plasmids had a similar size of approximately 91 kb. Comparison of the common regions among the 23 plasmid sequences identified only eight SNPs suggesting that the plasmids were very closely related. A phylogenetic tree of the plasmids showed five plasmid types with the majority (18 out of 23) belonging to one type with identical or near identical sequences (Fig. 3a). All plasmid-positive strains except four were clustered together on the core genome tree. Six plasmid positive strains, LM0429, LM0422, LM0476, LM0146, LM0099 and LM0208 were located on different branches of the tree, suggesting independent acquisitions of the plasmid.

### CRISPR systems in ST87 strains

The chromosome of strain ICDC-LM188 contains two CRISPR systems. Locus 1 is a remnant of a CRISPR system without any *cas* genes. The content of locus 1 showed 100% nucleotide identity with that of strain 1/2b SLCC2755 [23], and was conserved in all sequenced ST87 strains. The other CRISPR locus followed by *csn2, cas2, cas1* and *cas9*, which is inserted into the same location (*lmo2595*) as the reference strain EGD-e (Fig. 1). Complete CRISPR locus was only found in half of the sequenced ST87 strains (35/71), but the gene *cas9* was found in all sequenced strains. The incomplete CRISPR locus in some of the genomes is probably due to gaps in the genome assembly. For 35 ST87 strains with an intact CRISPR locus sequence, a total of 85 spacers and 22 unique spacer arrangements were observed, with spacer numbers between 29 and 40. The spacer content of the CRISPR locus in the ST87 strains was different from strains SLCC5850 (serotype 1/2a), SLCC2540 (serotype 3b), SLCC2755 (serotype 1/2b) and SLCC2482 (serotype 7), which had been reported by Kuenne et al [23]. The spacer profiles of each strain were presented in Fig. 2. CRISPR loci evolve via polarized addition of new spacers, or via internal deletion of spacers, or duplication of spacers to create variant profiles. The ST87 CRISPR profiles of spacer-distribution can be classified into two clusters, each of which has a major type represented by seven and nine strains, respectively. One minor type was represented by two strains, and the remaining types were represented by one strain.

### ST87-specific gene cluster

A ST87-specific gene cluster that harbored a type II restriction-modification (RM) system was identified and is present in all ST87 strains sequenced in this study (Fig. 1). The gene cluster was approximately 10 Kb in size encoding 10 open reading frames (ORFs) and inserted between the gene encoding homoserine dehydrogenase and the gene encoding 50S ribosomal protein L31 (Additional file3: Figure S3). The first ORF encodes a protein belonging to an AIPR superfamily which was identified as an abortive infection phage resistance protein often found in RM system operons [26]. The restriction endonuclease recognition site was predicted to be GCSGC (S=C or G) by BLASTN search using REBASE. The DNA methyltransferase, involved in attaching methyl group to C-5 cytosine, exhibited 86% nucleotide identity and 89% amino acid identity with a homologue in *Enterococcus faecium* strain T110 (locus tag: M395_10120). The GC content of the endonuclease gene, the methyltransferase gene and the whole gene cluster were 34.66, 33.16 and 33.78%, respectively, noticeably lower than the genome average of 38%. The gene cluster may have been acquired by horizontal gene transfer, probably from *Enterococcus faecium.*

### Virulence genes and other genetic elements in ST87 *L. monocytogenes* strains

All well-known virulence factors, including the *Listeria* pathogenicity island (LIPI)-1, *iap*, *fbp*A [27], *lpe*A [28], *lap* [29] and *lap*B [30] were found in all sequenced ST87 strains. All the sequenced ST87 strains contained multiple internalins, including internalin A, B, C, C2, D, E, F, I, J, and K. Also, there were no premature stop codons (PMSC) in *inlA* in any of the ST87 strains*.* PMSCs of *inl*A have often been found in *L. monocytogenes* isolates from food products but not human cases [20].

**Fig. 4:**
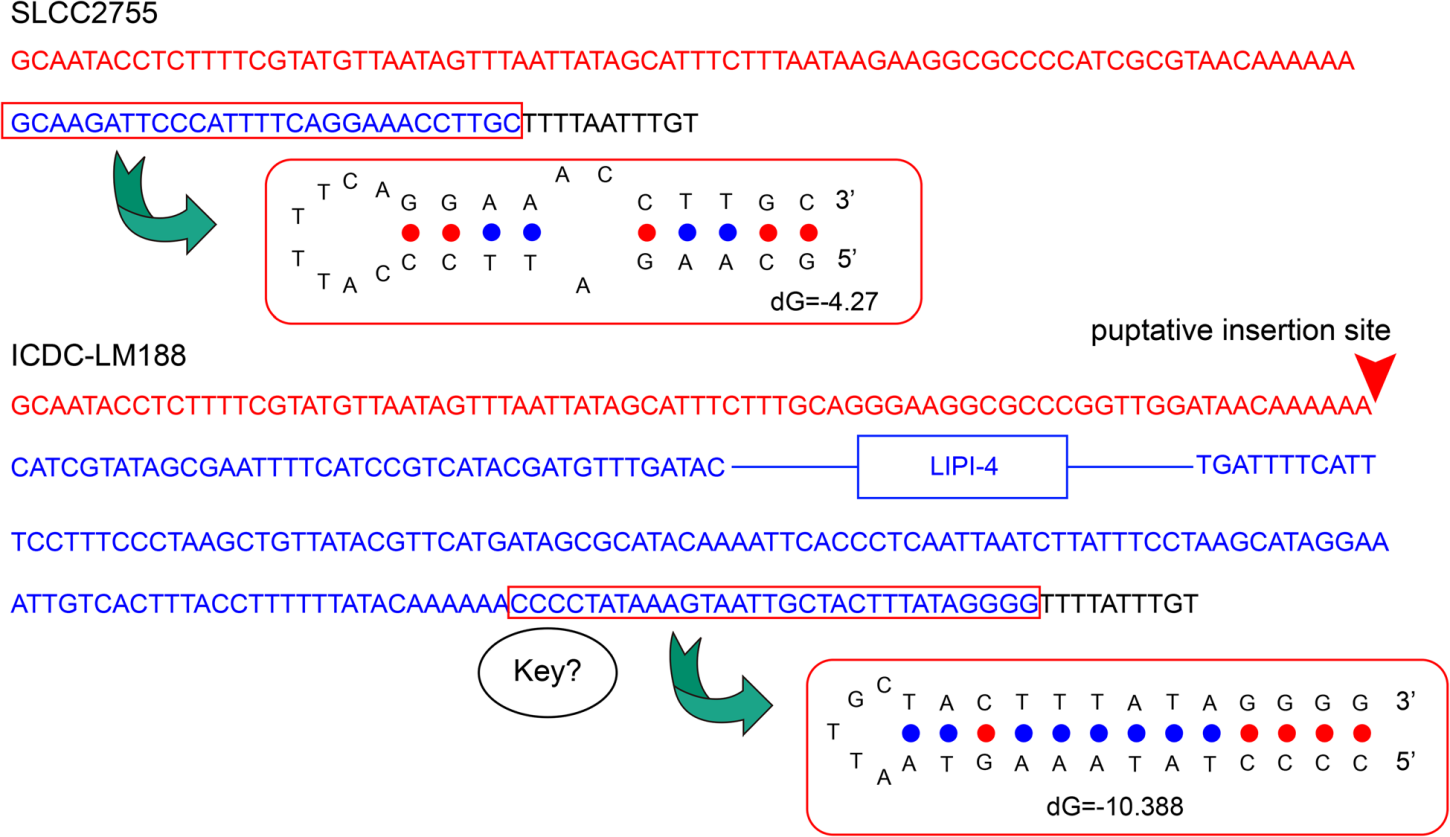
The flanking sequences of LIPI-4 in strain ICDC-LM188 (LIPI-4^+^) and the corresponding sequence in SLCC2755 (LIPI-4^−^). The identical sequences in both LIPI-4 positive and negative strains were shown in red (upstream) and blank (downstream). The part different was shown in blue. The stem-loop structures of the sequences framed in red were shown below

All ST87 strains studied harbored the newly reported LIPI-4 pathogenicity island [7], but not LIPI-3, Stress survival islet (SSI)-1 and SSI-2 [8–10]. Instead a LmoOf2365_0481 homolog is present at the location of SSI-1/SSI-2. The pairwise SNP differences in LIPI-4 among strain ICDC-LM188 (ST87), CLIP80459 (ST4), and CFSAN23463 (ST382) were eight or nine SNPs. The upstream flanking sequences of LIPI-4 in ICDC-LM188 and CFSAN23463 were the same, but differed by one SNP from CLIP80459, while the downstream flanking sequences among the three strains were identical. Interestingly, a lineage IV strain J1–208 also harbored LIPI-4, which differed by 223 SNPs from ICDC-LM188. The upstream and downstream sequences differed by 8 and 9 SNPs from ICDC-LM188, respectively (Additional file 4: Figure S4). The putative integration site of LIPI-4 was identified based on a 79-bp sequence with 95% nucleotide identity between LIPI-4^+^ strains and LIPI-4^−^ strains (the sequence marked in red in Fig. 4). In *Escherichia coli*, palindromes are sites of DNA double-strand break formations and the breaks can be repaired by RecBCD pathway through homologous recombination [31]. For LIPI-4^+^ *L. monocytogenes*, a perfect palindrome (the sequence framed in red in Fig. 4) was observed in the intergenic sequence between the LIPI-4 and its next gene, while for LIPI-4^−^ strains, an interrupted palindrome (the sequence boxed in blue in Fig. 4) was observed next to the putative integration site (Fig. 4). It has been suggested that the perfect palindrome might be the key to the acquisition of LIPI-4 [31].

## Discussion

In this study, 71 *L. monocytogenes* ST87 strains were sequenced to investigate the genetic features and relationships of strains of this prevalent ST in China. The 71 strains were divided into 10 clades. The clinical strains spread across multiple branches, suggesting that all ST87 strains can cause human infections. The mutation rate of *L. monocytogenes* ST87 was estimated to be 4.3 × 10^− 7^ substitutions per site per year which was similar to the estimate from a global *L. monocytogenes* study [32]. The Chinese ST87 strains did not cluster separately from non-Chinese ST87. The mixed-distribution of non-Chinese and Chinese ST87 strains suggests that ST87 has spread to other part of the world since it emerged, presumably, in China. This is not surprising as multiple globally distributed sublineages of *L. monocytogenes* have been found in the recent global *L. monocytogenes* study [32].

This study also included 10 strains from a one-year longitudinal investigation of *L. monocytogenes* contamination in retailing markets in Zigong, Sichuan province during 2014 to 2015 [15, 33]. Among the 10 strains from that study, five strains (LM1249, LM1459, LM1509, LM1605 and LM1637) were isolated from the same booth in February, April to July, 2015, respectively. While another five strains (LM1496, LM1514, LM1515, LM1523 and LM1542) were isolated from different booths which were located in the same market in May, 2015. All of the 10 strains were clustered into one clade (Clade 10) on the phylogenetic tree. Isolations of closely related strains over multiple sampling times in the same market indicated that this clone had persisted in the market environment and was likely to have served as the ultimate source of food contamination in the retail market.

Several previous genomic studies showed that genomic diversity of *L. monocytogenes* was mostly due to mobile genetic elements, such as prophages, transposons, and genetic islands [23]. Our study also identified prophages as the major source of genomic diversity in ST87 *L. monocytogenes*. A total of 11 prophage integration sites (Fig. 1), with 15 prophage profiles (Table 2) were identified. Prophages contributed to a significant proportion of ST87 accessory genome with 10 prophages variably present in different strains. Interestingly LM0097 has no prophages in its genome. It is interesting to note that LM0097 also has the least accessory genes, with 93.7% of the genomic genes belonging to the core. Prophages P2 and P3 were present in most strains (Fig. 2). The absence of P2 and P3 in some strains is probably due to loss of the prophages.

**Table 2:** The profiles of prophage insertion site in each ST87 *L. monocytogenes*

type	IS2 tRNA-Arg	IS3 comK	IS4 rumA	IS5 tsf	IS6 mat	IS7 50S rRNA methyltransferase	IS8 tRNA-Lys	IS9 tRNA-Ser	IS10 tRNA-Arg	IS11 tRNA-Thr	Strain list
1	+	+	–	–	–	–	–	–	–	–	ICDC-LM188, ICDC-LM0200, ICDC-LM0216, ICDC-LM0263, ICDC-LM0322, ICDC-LM0403, ICDC-LM0417, ICDC-LM0422, ICDC-LM0429, ICDC-LM0452, ICDC-LM0453, ICDC-LM0476, ICDC-LM0484, ICDC-LM0725, ICDC-LM0925, ICDC-LM1074, ICDC-LM1175, ICDC-LM1233, ICDC-LM1361, ICDC-LM1523, ICDC-LM1620, ICDC-LM1686
2	+	–	–	–	–	–	–	–	–	–	ICDC-LM0078, ICDC-LM0158, ICDC-LM544, ICDC-LM1459
3	–	+	–	–	–	–	–	–	–	–	ICDC-LM0077, ICDC-LM0143, ICDC-LM0159, ICDC-LM0250, ICDC-LM1197, ICDC-LM1249, ICDC-LM1496, ICDC-LM1509, ICDC-LM1541, ICDC-LM1515, ICDC-LM1534, ICDC-LM1542, ICDC-LM1551, ICDC-LM1605, ICDC-LM1637, ICDC-LM1784
4	+	+	–	–	–	–	–	–	+	–	ICDC-LM0007, ICDC-LM0053, ICDC-LM0111, ICDC-LM0658, ICDC-LM1203, ICDC-LM1204, ICDC-LM1220, ICDC-LM1572, ICDC-LM1674
5	+	–	–	–	–	–	–	–	+	–	ICDC-LM0099, ICDC-LM1296
6	–	+	–	–	–	–	–	–	+	–	ICDC-LM0138
7	+	+	–	+	–	–	–	–	+	–	ICDC-LM0146
8	–	–	+	–	–	–	–	–	–	–	ICDC-LM0106, ICDC-LM0108, ICDC-LM0336, ICDC-LM0915
9	–	+	+	–	–	–	–	–	–	–	ICDC-LM1016, ICDC-LM1117
10	+	+	–	–	+	–	–	–	–	–	ICDC-LM0441, ICDC-LM0449
11	+	+	–	–	–	–	+	–	–	–	ICDC-LM1520
12	–	+	–	–	–	+	–	–	–	–	ICDC-LM1681, ICDC-LM1682, ICDC-LM1685, ICDC-LM1689
13	–	+	–	–	–	–	–	+	–	–	ICDC-LM0402
14	–	–	–	–	–	–	–	–	–	+	ICDC-LM0208
15	–	–	–	–	–	–	–	–	–	–	ICDC-LM0097

Noteablely, P3 inserted in the *comK* site which is occupied by prophages A118 and ϕ10403S in other strains [34]. Further, excision of the prophage at the *comK* site plays an active role as a regulatory switch of *L. monocytogenes* in enhancing niche adaptation, and facilitating escape from phagosomes during mammalian cell infection [34–36]. P3 shared no homology with prophages, A118 and ϕ10403S, previously found in the *comK* site.

A conserved plasmid was present in 23 of the 71 ST87 *L. monocytogenes* strains. All of the plasmids from the ST87 strains in this study shared more than 99.9% sequence similarity to pLM1686 [16]. Plasmid pLM1686 belonged to *Listeria* plasmids *repA* group 2, which harbored large plasmids with sizes ranging from 55 kb to 100 kb [37]. The plasmid showed very closed relationship with pSHL004 from strain SHL004, which belonged to CC8 serotype 1/2a from China [38].

Most plasmid-containing strains (18/23) were derived from food products and environments from four retail markets during a 12-month longitudinal investigation in Zigong, Sichuan province [15]. It is possible that maintaining the plasmid may represent a competitive advantage for these strains. The plasmid contained cadmium resistance *cadAC* genes and also a putative multicopper oxidase gene and copper transporter gene *copB* [16]. A recent study of 3 plasmids, p4KSM, pLMR479a and pLM6179 from 3 different STs, ST5, ST8 and ST121, found that these plasmids contribute to tolerance against elevated temperature, salinity, acidic environments, oxidative stress and disinfectants [39]. These plasmids belonged to the same *repA* group 2 as the plasmids from our study. All three plasmids contained the cadmium resistance *cadAC* genes and one plasmid, pLMR479a, additionally carried a putative multicopper oxidase gene and copper transporter gene *copB* similar to pLM1686 [16]. Therefore, carriage of pLM1686 or pLM1686-like plasmid is likely to be advatantagous in surviving the envirotnment.

CRISPR/Cas system is a prokaryotic immune system that confers resistance to foreign DNA, e.g., a virus or plasmid. In the case of *Salmonella typhimurium*, the profile of spacer arrangements has strong association with strain phylogeny [40]. For ST87 strains, the CRISPR spacer patterns have no correlation with strain phylogeny. Two major types were observed. Strains carried one or the other major type but not both and the distribution of the major types was not clustered. For example, strains ICDC-LM0077 and ICDC-LM1074 were closely related (differing by 52 SNPs) but harbored entirely different sets of spacers. In contrast, strains ICDC-LM0097 and ICDC-LM0099 were distantly related to each other (differing by 294 SNPs), but harbored exactly the same set of spacers. There seem to be no phylogenetic patterns of the distribution of these two major CRISPR spacer types when compared with the genome tree.

Our study also shed light on the virulence and adaptation of ST87 at genomic level. A ST87 specific gene cluster that is conserved in all ST87 strains was found in this study which carried a novel RM system. RM systems have a diverse range of functions including playing a role in virulence and adaptation to hostile environments [41, 42]. Further studies are warranted to determine whether this novel RM system assists ST87 in survival in the environment and/or virulence in humans. All ST87 strains also carried LIPI-4, a cluster of six genes encoding a cellobiose-family phosphotransferase system (PTS), which has been identified as a virulence factor implicated in maternal-neonatal (MN) and central nervous system (CNS) infections previously [7, 22]. ST87 is prevalent in causing human infections in China and the presence of LIPI-4 may have contributed to its prevalence, which underscores the importance of epidemiological surveillance of ST87 from food sources and human infections in China.

## Conclusion

ST87 *L. monocytogenes* strains were frequently isolated from food products, environments and human clinical infections in China. The ST87 core genome carried 2677 genes. All ST87 strains carried prophage P1 and variably 10 other prophages as a major component of the ST87 accessory genome. ST87 contained a novel type II RM system although its significance is unknown, and a virulent gene cluster LIPI-4. A novel plasmid was present in a small proportion of the ST87 strains and nearly all were from food products or environments from four retail markets from a single region. The plasmid may offer the host an advantage to compete and persist in certain environments. ST87 arose 200 years ago and expanded rapidly in its early evolution in China. This study has shed light on the genomic diversity, evolution, adaptation and pathogenicity of ST87, the predominant ST that causes human infections in China.

## Methods

### Strains used for genome sequencing and DNA isolation

A total of 71 ST87 *L. monocytogenes* strains were used. The isolates were obtained from 2001 to 2015 from 10 regions of China including nine isolates from human infections, 47 from food sources and 11 from the environment (Additional file 5: Table S1). The strains were stored in Brain Heart Infusion (BHI) broth containing 15% glycerol at − 80 °C, and cultured on BHI agar at 37 °C. Genomic DNA from pure cultures was extracted using the Wizard® Genomic DNA Purification Kit (cat. # A1120, Promega, USA), according to the manufacturer’s instructions for Gram-positive bacteria.

### Genome sequencing, assembly and annotation

The whole genome of *L. monocytogenes* strain ICDC-LM188 was determined by using the Illumina HiSeq 2000 platform. The reads were assembled de novo into a high-quality draft genome using Velvet (version 1.2.10) with default parameters and with an average 85-fold sequencing coverage. All of the gaps were closed by primer walking and sequencing of PCR products using Applied Biosystems 3730 DNA Analyzer. Genome annotation was performed using the NCBI Prokaryotic Genomes Automatic Annotation Pipeline (PGAAP). Whole-genome shotgun sequencing of the other 70 ST87 *L. monocytogenes* strains was performed on the Illumina HiSeq X PE150 platform. The read quality was assessed with FastQC, and the reads were filtered to remove low-quality sequences (quality score ≤ 38), ambiguous reads (reads with 10 or more unknown base N) and adapter sequences. High-quality paired-end reads were assembled into scaffolds using SOAPdenovo v1.05. The coding genes were retrieved using the GeneMarkS (version 4.17, http://topaz.gatech.edu/)program [43] or Rapid Annotations based on Subsystem Technology (RAST) server (version 2, http://rast.nmpdr.org/rast.cgi) [44]. The database of Clusters of Orthologous Groups of proteins (COGs) was used to predict gene functions and the parameters for BLASTP searches were E-value of less than 10^− 5^ and larger than 40% of the query coverage.

### Core-pan genome analysis

Identification of homologous proteins was performed by the CD-HIT rapid clustering of similar proteins software [45], based on a similarity threshold of 50% amino acid identity and 70% coverage.

### Detection of the core genome SNPs

For the detection of SNPs, Illumina reads from the 70 ST87 strains sequenced were mapped against the closed genome of *L. monocytogenes* strain ICDC-LM188 using Burrows-Wheeler Aligner (BWA-MEM) [46]. SNP calls were performed by SAMtool (version 0.1.19) followed by BCFtools 1.9, and a vcf file for each isolate was generated. High-quality SNPs were identified using the following criteria: i). a minimum coverage of 20 reads; and ii). minimum variant frequency of 70% [47]. We searched against the COG database to catagorise the core genes of ST87.

### Phylogenetic analysis based on the whole genomic SNPs

The SNPs located in the core genome were used to perform phylogenetic analysis on all sequenced *L. monocytogenes* ST87 strains. The SNPs for each genome was concatenated to a multi-alignment FASTA file, and then imported to MEGA6 to generate a maximum likelihood tree using default parameters. For further evolutionary analysis of ST87 strains, phylogenetic inference was performed with Bayesian Evolutionary Analysis by Sampling Trees (BEAST, v2.4.7). BEAST analysis was performed with three replicates. The best-fit evolutionary analysis was performed under GTR model of substitution, along with a relaxed clock log normal clock model and Birth Death Model tree prior. We performed the run for 50,000,000 generations, sampling every 1000 generations and analyzed the output by using the Tracer module with all ESS values > 200. The burnin percentage was set to 10 for Markov chain Monte Carlo (MCMC) output analysis by TreeAnnotator (v2.4.7).

### Analysis of prophage, plasmids, CRISPR, LIPI-4 and virulence genes

Prophage regions were identified using PHASTER with default parameters online (http://phaster.ca) and were considered to be incomplete, questionable or complete when its completeness score was less than 60, between 60 and 90 or more than 90, respectively [48]. The plasmids in ST87 strains were identified by BLASTN searches using the sequence of pLM1686 as query against each assembled sequence. For the comparative analysis of pLM1686 with other *L. monocytogenes* plasmids, RepA protein sequences were used to infer their phylogenetic relationships by the Maximum Likelihood method. The CRISPR system was identified by CRISPRFinder from https://crispr.i2bc.paris-saclay.fr/Server/ [49]. The divergence of LIPI-4 and flanking sequences in *L. monocytogenes* ICDC-LM188, CFSAN023463, CLIP80459 and J1–208 was determined using MAUVE and BLASTN, respectively. The presence/absence of LIPI-3, stress survival islets SSI-1 and SSI-2 and other virulence genes were determined using BLASTN with a threshold of ≥60% query coverage with ≥80% nucleic acid sequence identity.

## Supplementary information

**Additional file 1: Figure S1.** COG analysis of core genes (A) and accessory genes (B). COG categories are as follows: C: Energy production and conversion, D: Cell cycle control, cell division, chromosome partitioning, E: Amino acid transport and metabolism, F: Nucleotide transport and metabolism, G: Carbohydrate transport and metabolism; H: Coenzyme transport and metabolism, I: Lipid transport and metabolism, J: Translation, ribosomal structure and biogenesis, L: Transcription, K: Replication, recombination and repair, M: Cell wall/membrane/envelope biogenesis, N: Cell motility, O: Posttranslational modification, protein turnover, chaperones, P: Inorganic ion transport and metabolism, Q: Secondary metabolites biosynthesis, transport and catabolism, R: General function prediction only; S: Function unknown; T: Signal transduction mechanisms; U: Intracellular trafficking, secretion, and vesicular transport; V: Defense mechanisms, W: Extracellular structures, X: Mobilome, prophages, transposons

**Additional file 2: Figure S2.** Maximum likelihood phylogenetic tree of Chinese and non-Chinese ST87 strains. The phylogenetic analysis was performed based on the SNPs located in the core genes of ST87 *L. monocytogenes*. The non-Chinese strains were colored blue.

**Additional file 3: Figure S3.** ST87-specific gene cluster. The putative function of each ORF is shown. The flanking genes of the cluster are in green. The two major components of the R-M system coding genes are in blue, while the other genes of this cluster are in red.

**Additional file 4: Figure S4.** The alignments of flanking intergenic sequences of LIPI-4. A for upstream region (located in ICDC-LM188 from 2,440,881 to 2,441,001) and B for downstream region (located in ICDC-LM188 from 2,447,012 to 2,447,174). *L. monocytogenes* strains ICDC-LM188 (ST87, Lineage I), CFSAN023463 (ST382, Lineage I), CLIP80459 (ST4, Lineage I) and J1–208 (ST unknown, Lineage IV).

**Additional file 5: Table S1.** List of ST87 L. monocytogenes strains used in this study.

**Additional file 6: Table S2.** Summary statistics of whole genome assemblies and gene prediction.

## Data Availability

The datasets generated and/or analysed during the current study are included in this published article. The genome sequences are deposited in NCBI database. The complete genome ICDC-LM188 has been deposited in DDBJ/ENA/GenBank under the accession number CP015593. The draft genome sequences of the other strains sequenced in this study has been deposited at DDBJ/ENA/Genbank under the BioProject accession number PRJNA447903. The accession numbers of LM1686 and pLM1686 are SAMN10373576 and MK134858, respectively. Publicly available raw sequence data of seven non-Chinese ST87 strains were included in the comparative analysis between the non-Chinese and Chinese ST87 *L. monocytogenes.* Six genomes were from NCBI’s SRA database with accession numbers for six sequences are SRR1112090, SRR1112227, SRR946037, SRR972401, SRR972405, SRR974856 and one was obtained from ENA with the accession number ERS1044951.

## Abbreviations

CC: Clonal complex
LIPI: *Listeria* pathogenicity island
MLST: Multi-locus sequence typing
RM system: Restriction-modification system
SNP: Single nucleotide polymorphism
SSI: Stress survival islet
ST: Sequence type
WGS: Whole genome sequencing
