# Supplementary figures and images for: Genomic dissection of the most prevalent Listeria monocytogenes clone, sequence type ST87, in China
Source: BMC Genomics. 2019 Dec 23;20:1014. doi: 10.1186/s12864-019-6399-1 (PMC6929445; doi:10.1186/s12864-019-6399-1)

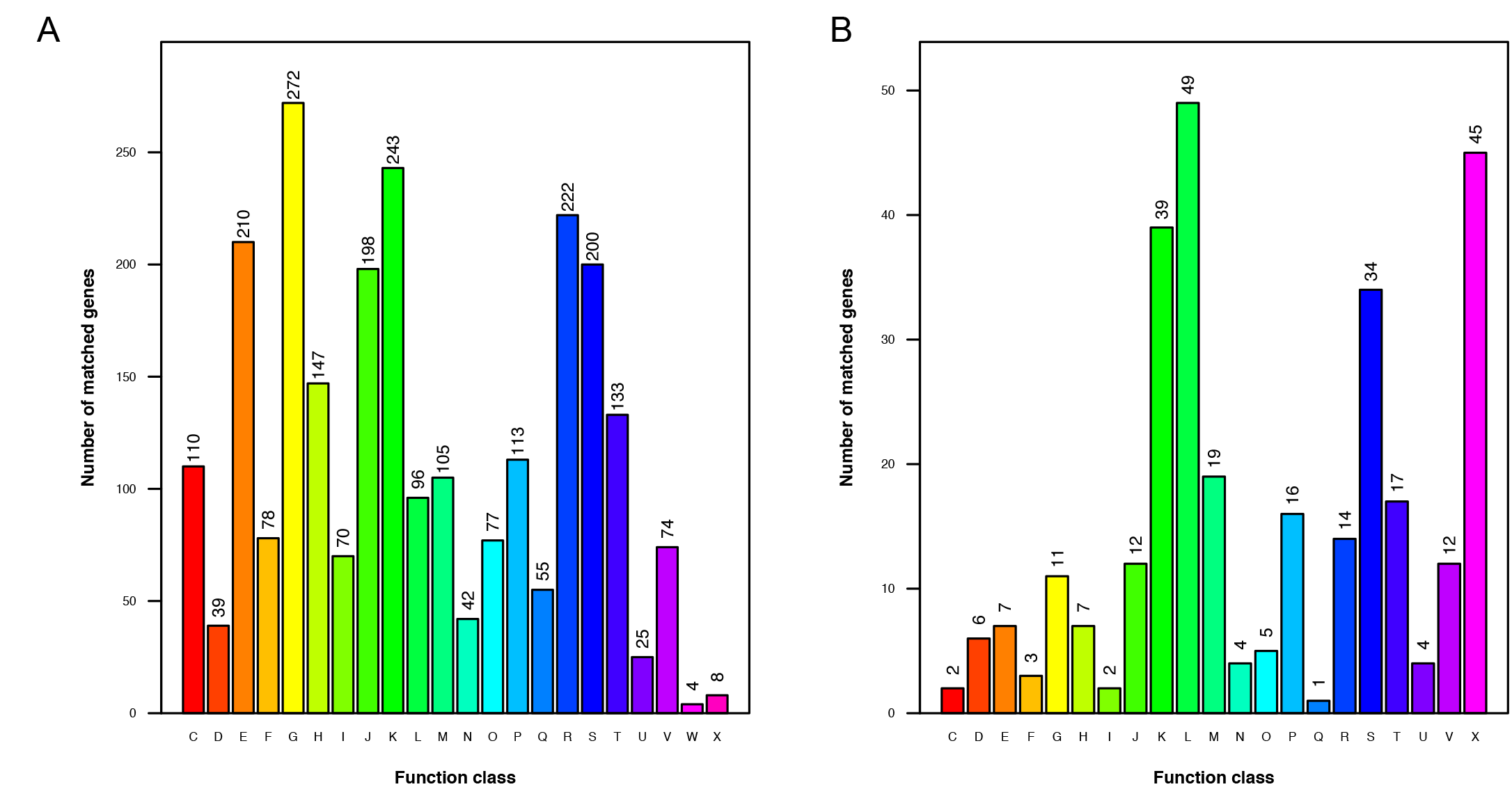

Supplement: Supplementary file 1 — Additional file 1: Figure S1. COG analysis of core genes (A) and accessory genes (B). COG categories are as follows: C: Energy production and conversion, D: Cell cycle control, cell division, chromosome partitioning, E: Amino acid transport and metabolism, F: Nucleotide transport and metabolism, G: Carbohydrate transport and metabolism; H: Coenzyme transport and metabolism, I: Lipid transport and metabolism, J: Translation, ribosomal structure and biogenesis, L: Transcription, K: Replication, recombination and repair, M: Cell wall/membrane/envelope biogenesis, N: Cell motility, O: Posttranslational modification, protein turnover, chaperones, P: Inorganic ion transport and metabolism, Q: Secondary metabolites biosynthesis, transport and catabolism, R: General function prediction only; S: Function unknown; T: Signal transduction mechanisms; U: Intracellular trafficking, secretion, and vesicular transport; V: Defense mechanisms, W: Extracellular structures, X: Mobilome, prophages, transposons [file 12864_2019_6399_MOESM1_ESM.tif]

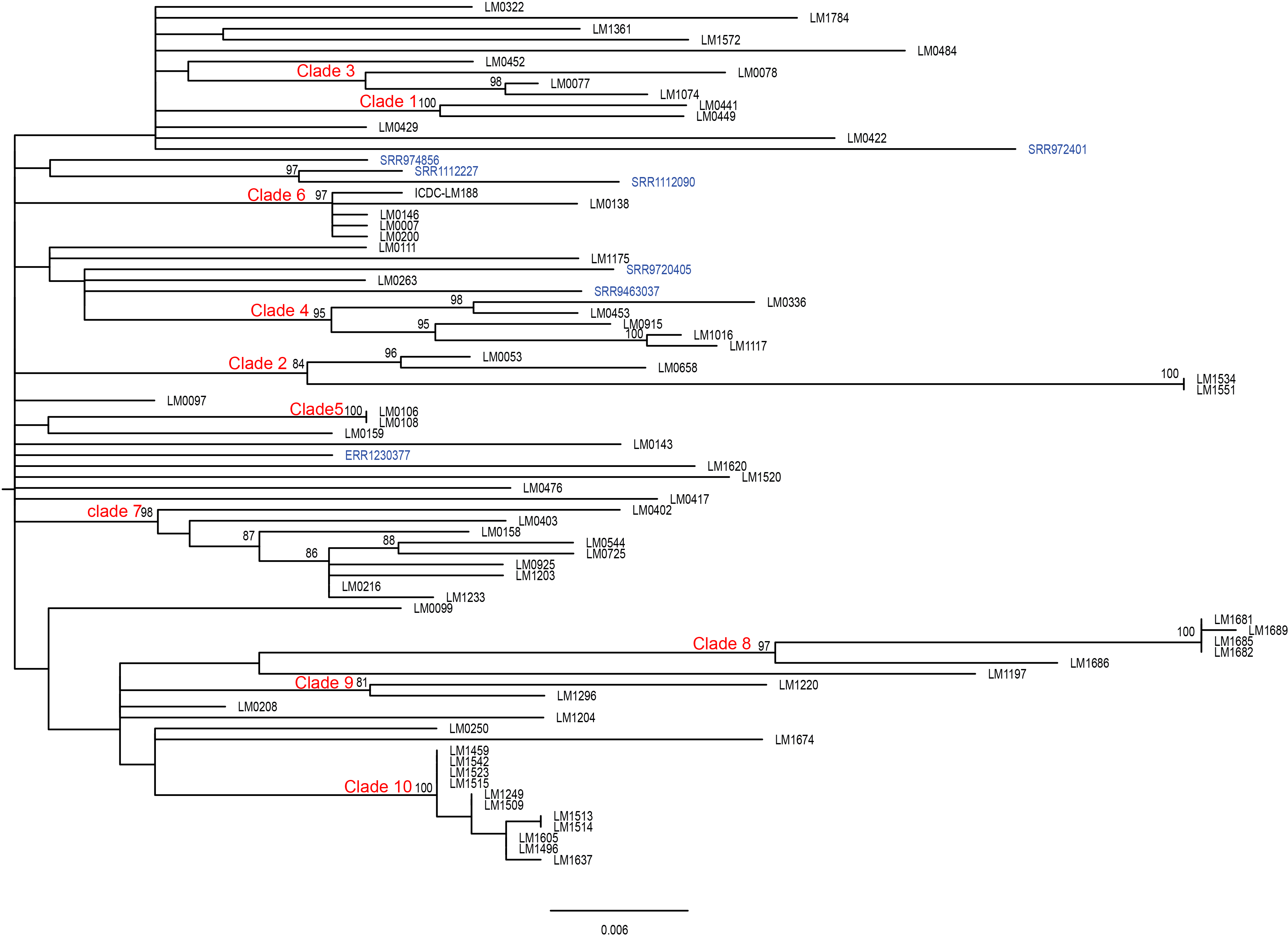

Supplement: Supplementary file 2 — Additional file 2: Figure S2. Maximum likelihood phylogenetic tree of Chinese and non-Chinese ST87 strains. The phylogenetic analysis was performed based on the SNPs located in the core genes of ST87 L. monocytogenes. The non-Chinese strains were colored blue. [file 12864_2019_6399_MOESM2_ESM.tif]

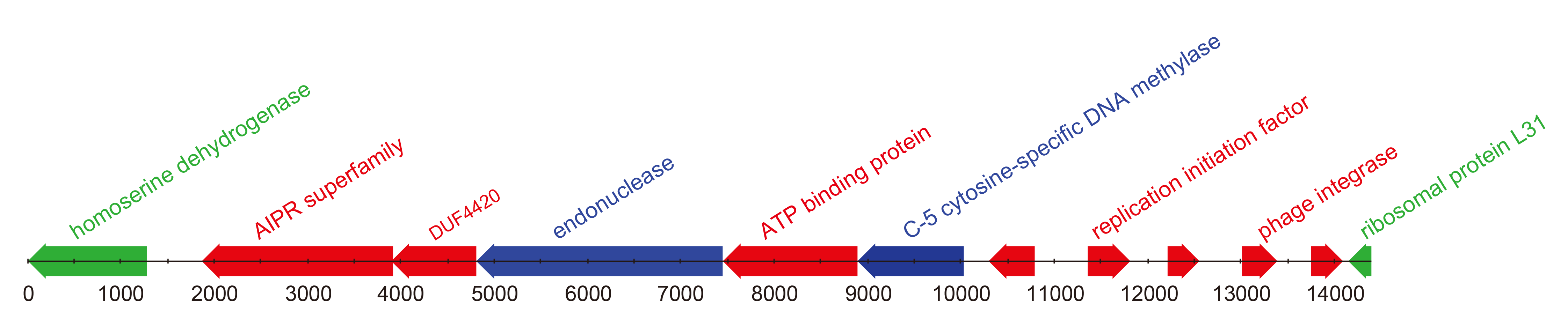

Supplement: Supplementary file 3 — Additional file 3: Figure S3. ST87-specific gene cluster. The putative function of each ORF is shown. The flanking genes of the cluster are in green. The two major components of the R-M system coding genes are in blue, while the other genes of this cluster are in red. [file 12864_2019_6399_MOESM3_ESM.tif]

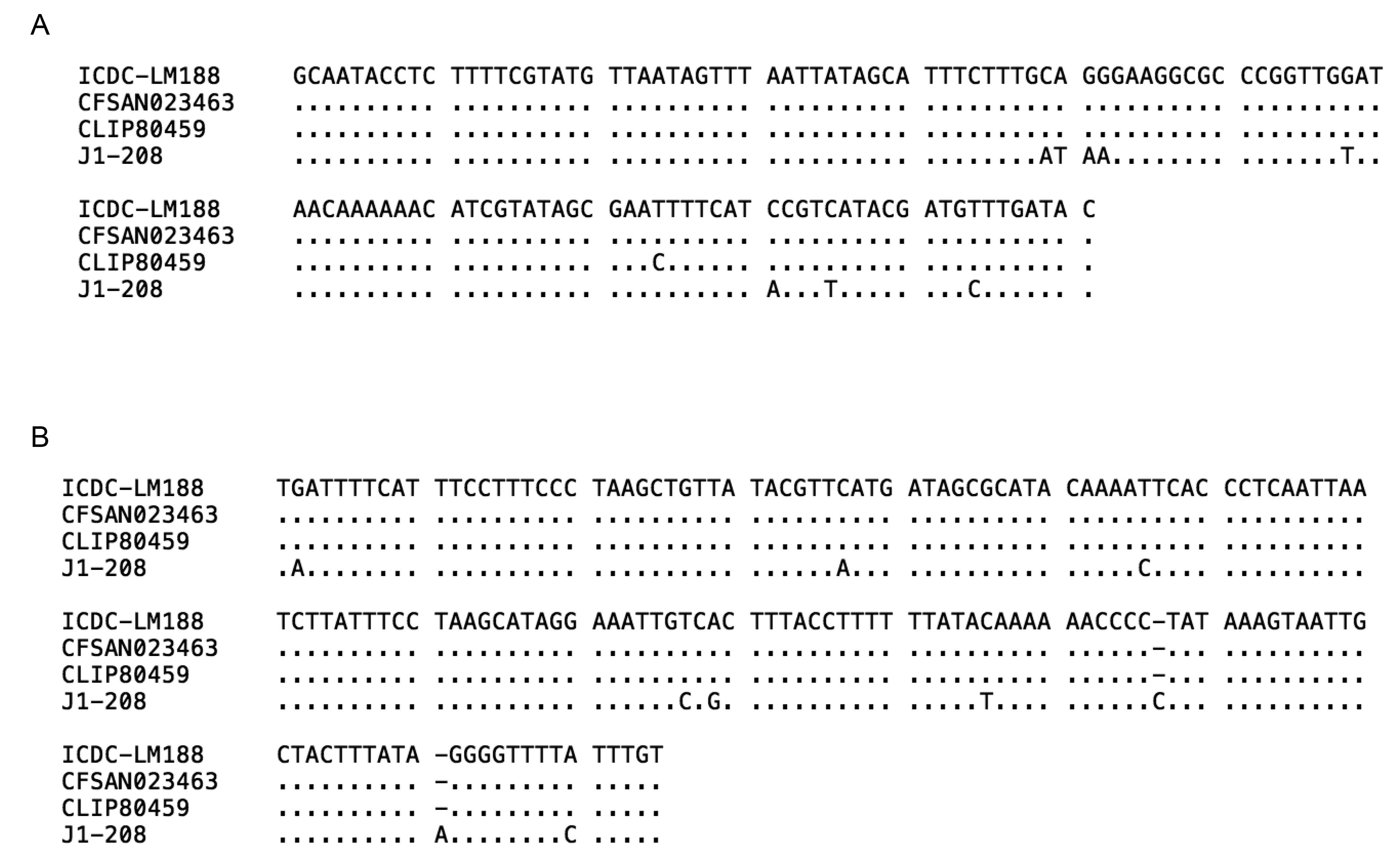

Supplement: Supplementary file 4 — Additional file 4: Figure S4. The alignments of flanking intergenic sequences of LIPI-4. A for upstream region (located in ICDC-LM188 from 2,440,881 to 2,441,001) and B for downstream region (located in ICDC-LM188 from 2,447,012 to 2,447,174). L. monocytogenes strains ICDC-LM188 (ST87, Lineage I), CFSAN023463 (ST382, Lineage I), CLIP80459 (ST4, Lineage I) and J1–208 (ST unknown, Lineage IV). [file 12864_2019_6399_MOESM4_ESM.tif]
