# Supplementary material for: Genomic dissection of the most prevalent Listeria monocytogenes clone, sequence type ST87, in China
Source: BMC Genomics. 2019 Dec 23;20:1014. doi: 10.1186/s12864-019-6399-1 (PMC6929445; doi:10.1186/s12864-019-6399-1)
Supplement: Supplementary file 5 — Additional file 5: Table S1. List of ST87 L. monocytogenes strains used in this study. [file 12864_2019_6399_MOESM5_ESM.docx]

Table S1. List of ST87 *L. monocytogenes* strains used in this study

| strain_ID | isolation year | region | source |
| --- | --- | --- | --- |
| ICDC-LM188 | 2008 | Beijing | patient |
| LM0007 | 2002 | Beijing | cooked food |
| LM0053 | 2001 | Henan | cooked food |
| LM0077 | 2001 | Beijing | raw meat |
| LM0078 | 2001 | Beijing | poultry |
| LM0097 | 2001 | Fujian | poultry |
| LM0099 | 2001 | Fujian | cooked food |
| LM0106 | 2001 | Zhejiang | cooked food |
| LM0108 | 2005 | Zhejiang | cooked food |
| LM0111 | 2002 | Zhejiang | raw meat |
| LM0138 | 2003 | Fujian | raw meat |
| LM0143 | 2003 | Fujian | raw meat |
| LM0146 | 2004 | Fujian | raw meat |
| LM0158 | 2005 | Guangdong | raw meat |
| LM0159 | 2006 | Guangdong | poultry |
| LM0200 | 2005 | Hubei | unknown |
| LM0208 | 2005 | Hubei | unknown |
| LM0216 | 2006 | Hubei | unknown |
| LM0250 | 2007 | Sichuan | cooked food |
| LM0263 | 2010 | Jiangsu | patient |
| LM0322 | 2009 | Zhejiang | poultry |
| LM0336 | 2011 | Beijing | patient |
| LM0402 | 2009 | Beijing | cooked food |
| LM0403 | 2010 | Beijing | patient |
| LM0417 | 2011 | Shanghai | patient |
| LM0422 | 2010 | Anhui | vegetable |
| LM0429 | 2011 | Anhui | aquatic food |
| LM0441 | 2008 | Shanghai | aquatic food |
| LM0449 | 2009 | Shanghai | raw meat |
| LM0452 | 2010 | Shanghai | raw meat |
| LM0453 | 2011 | Shanghai | raw meat |
| LM0476 | 2011 | Sichuan | unknown |
| LM0484 | 2012 | Anhui | cooked food |
| LM0544 | 2014 | Beijing | poultry |
| LM0658 | 2014 | Beijing | aquatic food |
| LM0725 | 2014 | Beijing | raw meat |
| LM0915 | 2014 | Beijing | poultry |
| LM0925 | 2014 | Beijing | raw meat |
| LM1016 | 2014 | Beijing | environment |
| LM1074 | 2015 | Beijing | poultry |
| LM1117 | 2015 | Beijing | aquatic food |
| LM1175 | 2014 | Sichuan | raw meat |
| LM1197 | 2014 | Sichuan | raw meat |
| LM1203 | 2015 | Sichuan | aquatic food |
| LM1204 | 2015 | Sichuan | aquatic food |
| LM1220 | 2015 | Sichuan | environment |
| LM1233 | 2015 | Sichuan | poultry |
| LM1249 | 2015 | Sichuan | environment |
| LM1296 | 2015 | Sichuan | raw meat |
| LM1361 | 2015 | Sichuan | cooked food |
| LM1459 | 2015 | Sichuan | environment |
| LM1496 | 2015 | Sichuan | raw meat |
| LM1509 | 2015 | Sichuan | environment |
| LM1513 | 2015 | Sichuan | raw meat |
| LM1514 | 2015 | Sichuan | raw meat |
| LM1515 | 2015 | Sichuan | raw meat |
| LM1520 | 2015 | Sichuan | aquatic food |
| LM1523 | 2015 | Sichuan | raw meat |
| LM1534 | 2015 | Sichuan | environment |
| LM1542 | 2015 | Sichuan | raw meat |
| LM1551 | 2015 | Sichuan | environment |
| LM1572 | 2015 | Sichuan | cooked food |
| LM1605 | 2015 | Sichuan | environment |
| LM1620 | 2015 | Sichuan | environment |
| LM1637 | 2015 | Sichuan | environment |
| LM1674 | 2015 | Sichuan | cooked food |
| LM1681 | 2015 | Sichuan | patient |
| LM1682 | 2015 | Sichuan | patient |
| LM1685 | 2015 | Sichuan | patient |
| LM1689 | 2015 | Sichuan | patient |
| LM1784 | 2015 | Sichuan | environment |
