# Supplementary material for: Genomic dissection of the most prevalent Listeria monocytogenes clone, sequence type ST87, in China
Source: BMC Genomics. 2019 Dec 23;20:1014. doi: 10.1186/s12864-019-6399-1 (PMC6929445; doi:10.1186/s12864-019-6399-1)
Supplement: Supplementary file 6 — Additional file 6: Table S2. Summary statistics of whole genome assemblies and gene prediction. [file 12864_2019_6399_MOESM6_ESM.docx]

Table S2. Summary statistics of whole genome assemblies and gene prediction

| Strain | Scaffold_No. | Total Length(bp) | N50 Length(bp) | Max Length(bp) | Min Length(bp) | GC% | Gene No. | Gene average length(bp) |
| --- | --- | --- | --- | --- | --- | --- | --- | --- |
| LM0007 | 26 | 3,002,072 | 481,810 | 791,029 | 758 | 37.82 | 3,016 | 890 |
| LM0053 | 23 | 2,999,033 | 544,932 | 1,018,005 | 708 | 37.87 | 3,019 | 894 |
| LM0077 | 13 | 2,926,924 | 585,732 | 982,631 | 684 | 37.9 | 2,918 | 903 |
| LM0078 | 15 | 2,970,806 | 562,214 | 1,022,539 | 566 | 37.88 | 2,986 | 896 |
| LM0097 | 13 | 2,886,525 | 562,191 | 982,628 | 566 | 37.93 | 2,854 | 911 |
| LM0099 | 16 | 3,063,366 | 562,214 | 1,021,053 | 563 | 37.85 | 3,090 | 892 |
| LM0106 | 14 | 2,942,037 | 478,579 | 862,844 | 566 | 37.98 | 2,912 | 909 |
| LM0108 | 13 | 2,939,459 | 562,280 | 983,069 | 566 | 37.97 | 2,915 | 910 |
| LM0111 | 23 | 3,006,615 | 543,144 | 1,027,736 | 563 | 37.84 | 3,030 | 892 |
| LM0138 | 19 | 2,954,767 | 543,232 | 1,461,999 | 745 | 37.88 | 2,942 | 894 |
| LM0143 | 12 | 2,923,951 | 600,456 | 982,629 | 566 | 37.91 | 2,917 | 904 |
| LM0146 | 26 | 3,138,782 | 529,168 | 1,016,614 | 758 | 37.81 | 3,187 | 878 |
| LM0158 | 13 | 2,943,187 | 479,180 | 906,057 | 566 | 37.91 | 2,933 | 902 |
| LM0159 | 13 | 2,925,965 | 600,456 | 982,653 | 563 | 37.91 | 2,914 | 905 |
| LM0200 | 17 | 2,278,124 | 504,888 | 1,017,964 | 567 | 37.88 | 2,963 | 896 |
| LM0208 | 12 | 3,007,564 | 562,214 | 982,610 | 708 | 37.89 | 2,998 | 904 |
| LM0216 | 15 | 2,981,328 | 521,901 | 985,995 | 563 | 37.87 | 2,989 | 900 |
| LM0250 | 13 | 2,928,102 | 602,276 | 982,601 | 566 | 37.91 | 2,920 | 902 |
| LM0263 | 12 | 2,971,656 | 602,033 | 1,027,147 | 566 | 37.88 | 2,986 | 897 |
| LM0322 | 14 | 2,963,543 | 600,105 | 1,021,422 | 684 | 37.89 | 2,965 | 899 |
| LM0336 | 14 | 2,943,156 | 562,190 | 982,630 | 539 | 37.97 | 2,918 | 909 |
| LM0402 | 14 | 2,983,666 | 479,403 | 602,404 | 953 | 37.86 | 2,997 | 895 |
| LM0403 | 13 | 2,981,756 | 602,428 | 1,024,605 | 566 | 37.86 | 2,988 | 899 |
| LM0417 | 14 | 2,971,724 | 529,347 | 1,018,946 | 566 | 37.87 | 2,983 | 896 |
| LM0422 | 18 | 3,061,945 | 549,453 | 985,093 | 566 | 37.85 | 3,078 | 895 |
| LM0429 | 23 | 3,058,747 | 253,515 | 765,024 | 604 | 37.85 | 3,067 | 893 |
| LM0441 | 15 | 3,016,616 | 524,985 | 1,017,295 | 566 | 37.89 | 3,063 | 887 |
| LM0449 | 18 | 3,012,624 | 521,931 | 1,017,207 | 539 | 37.88 | 3,063 | 886 |
| LM0452 | 15 | 2,969,907 | 548,908 | 985,084 | 539 | 37.87 | 2,982 | 898 |
| LM0453 | 13 | 2,970,761 | 602,802 | 1,030,107 | 566 | 37.88 | 2,980 | 897 |
| LM0476 | 14 | 3,058,589 | 602,106 | 1,022,730 | 539 | 37.84 | 3,080 | 894 |
| LM0484 | 18 | 2,965,198 | 524,304 | 985,494 | 566 | 37.87 | 2,966 | 900 |
| LM0544 | 14 | 2,943,274 | 478,579 | 905,260 | 563 | 37.89 | 2,936 | 902 |
| LM0658 | 45 | 3,034,233 | 478,579 | 564,672 | 527 | 37.82 | 3,057 | 887 |
| LM0725 | 17 | 2,979,660 | 602,398 | 1,024,585 | 569 | 37.85 | 2,992 | 897 |
| LM0915 | 12 | 2,942,238 | 513,776 | 863,083 | 708 | 37.97 | 2,911 | 911 |
| LM0925 | 15 | 2,984,073 | 602,434 | 905,985 | 566 | 37.86 | 2,993 | 896 |
| LM1016 | 14 | 2,981,115 | 599,909 | 982,597 | 566 | 37.96 | 2,971 | 904 |
| LM1074 | 16 | 2,970,021 | 524,696 | 1,017,148 | 566 | 37.86 | 2,983 | 895 |
| LM1117 | 16 | 2,979,756 | 356,025 | 731,464 | 566 | 37.96 | 2,966 | 903 |
| LM1175 | 13 | 2,970,132 | 600,444 | 906,773 | 563 | 37.88 | 2,983 | 896 |
| LM1197 | 18 | 3,012,605 | 511,408 | 862,844 | 708 | 37.88 | 3,004 | 900 |
| LM1203 | 25 | 3,022,560 | 602,419 | 983,777 | 566 | 37.83 | 3,050 | 887 |
| LM1204 | 22 | 3,095,214 | 562,214 | 1,016,855 | 566 | 37.83 | 3,132 | 888 |
| LM1220 | 44 | 3,121,460 | 478,578 | 983,838 | 511 | 37.79 | 3,164 | 882 |
| LM1233 | 17 | 2,981,891 | 602,422 | 1,026,337 | 565 | 37.86 | 2,995 | 897 |
| LM1249 | 16 | 3,017,740 | 602,276 | 982,643 | 566 | 37.88 | 3,022 | 898 |
| LM1296 | 24 | 3,100,549 | 478,753 | 983,073 | 515 | 37.81 | 3,150 | 885 |
| LM1361 | 16 | 2,971,647 | 521,572 | 1,021,482 | 566 | 37.87 | 2,987 | 895 |
| LM1459 | 15 | 3,016,436 | 602,276 | 982,601 | 566 | 37.87 | 3,017 | 899 |
| LM1496 | 16 | 3,016,038 | 478,579 | 879,136 | 566 | 37.87 | 3,021 | 898 |
| LM1509 | 15 | 3,017,509 | 602,276 | 982,601 | 566 | 37.88 | 3,022 | 898 |
| LM1514 | 16 | 3,017,380 | 602,276 | 982,583 | 566 | 37.88 | 3,021 | 899 |
| LM1515 | 15 | 3,018,874 | 479,179 | 863,075 | 566 | 37.87 | 3,022 | 897 |
| LM1520 | 20 | 3,002,789 | 538,550 | 1,025,009 | 566 | 37.84 | 3,039 | 890 |
| LM1523 | 14 | 3,061,528 | 585,557 | 1,024,557 | 563 | 37.85 | 3,087 | 892 |
| LM1534 | 20 | 2,911,185 | 325,856 | 425,001 | 953 | 37.91 | 2,890 | 907 |
| LM1542 | 14 | 3,020,280 | 602,276 | 1,461,868 | 563 | 37.87 | 3,024 | 900 |
| LM1551 | 10 | 2,915,993 | 599,906 | 982,652 | 953 | 37.93 | 2,895 | 908 |
| LM1572 | 39 | 3,118,660 | 505,800 | 1,021,366 | 507 | 37.82 | 3,157 | 887 |
| LM1605 | 18 | 3,016,865 | 478,757 | 602,276 | 541 | 37.88 | 3,022 | 897 |
| LM1620 | 12 | 2,958,552 | 602,177 | 1,025,082 | 538 | 37.89 | 2,974 | 898 |
| LM1637 | 15 | 3,019,218 | 602,276 | 982,643 | 563 | 37.87 | 3,024 | 899 |
| LM1674 | 27 | 3,091,352 | 546,414 | 1,026,438 | 566 | 37.82 | 3,128 | 888 |
| LM1681 | 36 | 2,977,252 | 478,579 | 621,404 | 501 | 37.86 | 2,975 | 895 |
| LM1682 | 30 | 2,978,609 | 478,579 | 630,416 | 566 | 37.87 | 2,979 | 894 |
| LM1685 | 30 | 2,981,115 | 478,579 | 621,412 | 796 | 37.86 | 2,981 | 895 |
| LM1689 | 31 | 2,975,484 | 478,408 | 630,416 | 708 | 37.86 | 2,973 | 896 |
| LM1784 | 16 | 3,040,149 | 478,579 | 879,146 | 566 | 37.93 | 3,035 | 902 |
